# Genotoxic Potential of Thymol on Honey Bee DNA in the Comet Assay

**DOI:** 10.3390/insects14050451

**Published:** 2023-05-11

**Authors:** Uroš Glavinić, Milan Rajković, Marko Ristanić, Jevrosima Stevanović, Branislav Vejnović, Ninoslav Djelić, Zoran Stanimirović

**Affiliations:** 1 Department of Biology, Faculty of Veterinary Medicine, University of Belgrade, 11000 Belgrade, Serbia; 2 Department of Economics and Statistics, Faculty of Veterinary Medicine, University of Belgrade, 11000 Belgrade, Serbia

**Keywords:** *Apis mellifera*, continuous cell line, single cell gel electrophoresis, essential oils extracts, DNA damage

## Abstract

**Simple Summary:**

The aim of the present study was to evaluate, for the first time, the genotoxic and antigenotoxic potential of thymol on the honey bee continuous cell line AmE-711 using the Comet assay. Thymol did not show antigenotoxic effect on honey bee cells in any of the tested concentrations (10, 100, and 1000 μg/mL). Two concentrations (100 and 1000 μg/mL) expressed genotoxic effects on cultured honey bee cells, thus suggesting the careful application of thymol in beekeeping practice to avoid possible negative effects on honey bees.

**Abstract:**

Thymol is a natural essential oil derived from the plant *Thymus vulgaris* L. It is known to be beneficial for human and animal health and has been used in beekeeping practice against *Varroa* mite for years. In this study, the genotoxic and antigenotoxic potential of thymol were evaluated on the honey bee (*Apis mellifera* L.) continuous cell line AmE-711 for the first time. Using the Comet assay, three increasing concentrations (10, 100, and 1000 µg/mL) of thymol were tested. Negative control (non-treated cells) and positive control (cells treated with 100 µM H_2_O_2_) were also included. The absence of thymol cytotoxicity was confirmed with the Trypan blue exclusion test. Thymol in the concentration of 10 µg/mL did not increase DNA damage in AmE-711 honey bee cells, while 100 and 1000 µg/mL concentrations showed genotoxic effects. For testing the antigenotoxic effect, all concentrations of thymol were mixed and incubated with H_2_O_2_. The antigenotoxic effect against was absent at all concentrations (10, 100, 1000 μg/mL) tested. Moreover, thymol enhanced the H_2_O_2_-induced DNA migration in the Comet assay. The obtained results indicate genotoxic effects of thymol on cultured honey bee cells suggesting its careful application in beekeeping practice to avoid possible negative effects on honey bees.

## 1. Introduction

One of the most destructive diseases of honey bees (*Apis mellifera* L.) is varroosis, caused by the bee mite (*Varroa destructor* Anderson and Trueman) [1,2]. *V. destructor* parasitizes in both adults and immature bees and causes problems by feeding on their body fat and haemolymph [3,4]. In addition to its direct negative effects on bees, *V. destructor* is a vector of numerous pathogens (honey bee viruses and possibly *Nosema ceranae* Fries) that cause serious diseases in bees [5,6,7,8]. The mite is considered to be one of the main causes of honey bee colony losses. Since there is no absolute effective treatment strategy for the control of varroosis, colony collapse occurs in cycles of 2–3 years [9,10].

Numerous acaricides are available for the treatment of *Varroa* mites ranging from synthetic, or “hard”, to naturally derived, or “soft”, agents [1,9]. The major disadvantages of synthetic acaricides are their toxic side effects on bee colonies and the occurrence of chemical residues in bees and their products [10,11,12]. Accumulation of acaricide residues in wax and other bee products may accelerate the development of resistance to *V. destructor*, due to prolonged exposure of parasites to low doses of acaricides [1]. It is known that mites can develop resistance to many pesticides within a few generations [13]; therefore, intensive and repeated use of the same pesticides can lead to the development of resistance in parasite populations [14,15,16,17]. Given the negative effects of synthetic acaricides mentioned above, natural compounds have been found to play a critical role in the treatment of *Varroa* mites. Natural-based (“soft”) acaricides are often prepared from the essential oils of plants containing one or more components as active ingredients with proven acaricidal properties [12,18]. Organic acids such as oxalic acid [19,20], formic acid [21], lactic acid [22], and many essential oils [23,24] including thymol [25,26] are frequently used “soft” acaricides.

Thymol is an essential plant oil derived from thyme (*Thymus vulgaris*) and commonly used in pharmacognosy [27]. In addition to thymol (10–64%), thyme has high concentrations of many monoterpene phenols, such as carvacrol (0.4–20.6%), p-cymene (9.1–22.2%), cineole (0.2–14.2%), linalool (2.2–4.8%), borneol (0.6–7.5%), α-pinene (0.9–6.6%), and camphor 0–7.3% [28,29,30]. Essential oils such as thymol and its derivatives are known to be used in food and agricultural industries, as well as in cosmetics and medicine [31]. In addition to its positive therapeutic effect in cases of disorders of the heart, blood vessels, lungs, nervous and digestive systems in mammals [32,33,34,35], positive antiparasitic and antimicrobial effects have also been reported in mammals and bees [34,36,37,38,39,40,41]. The inhibitory effect of thymol on the growth of pathogenic bacteria and fungi, such as *Salmonella typhimurium*, *Staphylococcus aureus*, *Aspergillus flavus* and *Cryptococcus neoformans*, has been known for years [36]. In beekeeping, it has been used for decades to control the bee mite *V. destructor* [41]. At the beginning of the 21st century, the first investigations into the potential effect of thymol in the control of *Nosema* infection in the hive were conducted [42,43]. Thymol is one of the few compounds that has been shown to reduce *N. ceranae* and/or *N*. *apis* spore loads and reduce mortality in *Nosema*-infected bees [36,38,39,40]. The findings of Glavinic et al. [38] show that, when given to *Nosema*-infected bees, thymol has mainly beneficial impacts on health (raising levels of immune-related genes and values of oxidative stress indicators, and decreasing *Nosema* spore burdens). 

However, there is sufficient evidence of the negative impact of thymol on bees in both laboratory and field types of experiments. In a laboratory, thymol was the most toxic of all tested monoterpenoids when applied on bees as a fumigant [44] and thyme essential oil (composed of 65.3% of thymol) was the only one essential oil that caused bee mortality after their exposure to topical, vapour, and potentially oral treatments of four types of botanical oils [23]. The adverse effects of thymol recorded in field conditions are more varied. In a field study on 40 honey bee colonies, thymol crystals (in a gauze bag) had negative effects on the brood; in fact, bees removed larvae and pupae when thymol bags had been placed near the brood [45]. Moreover, significant negative effects on colony development were recorded as a consequence of treatments with thymol-based formulations (ApiLife VAR^®^ and Apiguard^®^) in a study conducted by Floris et al. [25]. The same formulations (Apiguard^®^ and Apilife Var^®^) also affected the behaviour of adult bees under either laboratory or hive conditions [46,47,48]. Among the detrimental effects that can be caused by the use of thymol in field conditions, supersedure and loss of the queen are also reported [49]; as much as 50% of colonies lost the queen after thymol-oil spray treatments [50]. Interestingly, thymol acted differently on *Nosema*-infected and *Nosema*-free bees if we interpret its effect based on transcription levels of immune-related genes and values of oxidative stress parameters; only *Nosema*-free bees were negatively affected by thymol as their survival was compromised, while oxidative capacity and regulation of some immune-genes decreased [38]. Caution in using thymol in honey bee hives was recommended by both Glavinić et al. [38] and Glavan et al. [51], the latter due to their findings that the use of the lowest concentration, which is effective against *Varroa* mites, could still affect honey bees [51].

Evaluation of cytotoxic and genotoxic properties of thymol was carried out using several cell systems [52,53,54,55]; in these experiments, synthetic thymol-based preparations that are usually used in beekeeping practice were tested [56]. An earlier report performed on cultured Syrian hamster embryo cells suggests that thymol can cause morphological transformations, DNA damage, and sister chromatid exchanges [57]. Günes-Bayir et al. [54] showed that thymol has a dose-dependent genotoxic effect on gastric adenocarcinoma cells. In contrast, LLana-Ruiz-Cabello et al. [58] reported the absence of genotoxic effects of thymol in the human colon carcinoma cell line.

Even though thymol was tested in various in vitro tests on different cell lines using different protocols, it has never been tested on honey bee cells. Keeping in mind that thymol is widely used in beekeeping practice and that continuous honey bee cell line AmE-711 has recently been developed, our team was inspired to test thymol (anti) genotoxicity using the alkaline Comet assay. To our knowledge, this is the first study of thymol genotoxicity evaluation on a honey bee cell line.

## 2. Material and Methods

### 2.1. Origin and Maintenance of Cell Cultures

The continuous cell line derived from honey bee (*Apis mellifera* L.) AmE-711 was used for evaluation of genotoxicity and antigenotoxicity thanks to its creator Michael Goblirsch [59] and the Bee Research Facility, University of Minnesota, USA. Schneider’s Insect Medium (Sigma-Aldrich, St. Louis, MO, USA) enriched with 10% foetal bovine serum (Sigma) was used as maintaining medium, while cells were incubated at 32 °C.

### 2.2. Assessment of the Cytotoxicity of Thymol on Honey Bee Cells

The determination of the cytotoxicity of thymol was performed using the Trypan blue exclusion dye assay according to the described protocol [60]. A total of 25 μL of the cell suspension at a density of 1 × 10^6^ cells/mL were treated with selected concentrations of thymol in 1.5 mL mini tubes. Even though, different studies evaluated toxicity of thymol on bees [61] and bee larvae [62], we decided to test the concentration of 100 µg/mL of thymol based on previous studies [38,39] in which bees were fed perorally in order to evaluate antipathogen effect. In addition to this concentration, we evaluated 10× lower (10 µg/mL) and 10× higher (1000 µg/mL) concentration of thymol, as well. However, selected concentration of 100 µg/mL is much lower of those in commercially available thymol-based products [25]. The negative (PBS solution) and positive (100 µM H_2_O_2_) controls were used. Cells were incubated at 32 °C for 1 h. In total, 25 µL of cell suspension mixed with 725 µL of PBS were stained with 250 μL of 0.4% Trypan blue dye (Sigma-Aldrich, St. Louis, MO, USA) in 1.5 mL tubes. After a 5 min incubation, cells were counted in a Neubauer chamber and a proportion of dead (stained) and viable (non-stained) cells was expressed as the percentage of viable cells.

### 2.3. Preparation of Cells for the Comet Assay

Trypsinization and washing in PBS solution (centrifugation at 1800× *g* for 10 min) were performed on cells from the 61st passage. To achieve a final cell density of 1 × 10^6^ cells/mL, the washed cells were placed in 1.5 mL polypropylene tubes and diluted in 1 × PBS.

### 2.4. Treatment of Cells for the Comet Assay

For evaluating thymol’s genotoxic effects, five experimental sets of cells were established. In the negative control group, cells were treated only with the PBS solution, whereas the cells in the positive control group were treated with 100 µM H_2_O_2_. In three thymol-treated groups, the following experimental concentrations of thymol were tested: 10, 100 and 1000 µg/mL. For the purpose of the experiment, we used thymol with a purity of ≥98.5% (Sigma-Aldrich, St. Louis, MO, USA, Product Number T0501, CAS 89-83-8).

To determine possible antigenotoxic effects of thymol, in addition to negative (incubated in PBS only) and positive control (treated with 100 µM H_2_O_2_), three more groups were established in which AmE-711 cells were co-treated with 100 µM H_2_O_2_ and thymol in concentrations of 10 µg/mL (group P10), 100 µg/mL (group P100) and 1000 µg/mL (group P1000). All experimental groups were incubated for 30 min at 32 °C.

### 2.5. The Comet Assay on AmE-711 Honey Bee Cells

The Comet test was carried out in accordance with the method of Singh et al. [63] and Tice et al. [64] with a few minor adjustments for the AmE-711 honey bee cell line in accordance with the work of Rajkovic et al. [65]. The first step comprised a thorough wash of the microscopic slides using detergent, after which the slides were rinsed and immersed in ddH_2_O. Prewashed microscopic slides were then submerged in a tank filled with 96% ethanol for a minimum of 24 h. Further on, slides were sterilized on a laboratory alcohol burner and precoated with 1% agarose with a normal melting point and left to dry at room temperature for 48 h, making a first layer. Agarose gel needed for the first layer was made from 2.5 g normal melting point agarose mixed with 250 mL ddH_2_O and heated in a microwave oven for 1 min. For the second and third layers of the gel, 0.67% and 0.50% low melting point agarose—LMPA was prepared by mixing 0.067 g and 0.05 g of agarose, respectively, in 10 mL of 1 × PBS. These mixtures were heated on a thermal shaker at temperatures up to 50 °C.

Further on, low melting point agarose—LMPA (0.67%)—and 100 μL of the cells were mixed. This suspension (90 μL) was spread with a coverslip on microscopic slide and placed in the refrigerator (4 °C) for 5 min in order to make the second layer. After the removal of the coverslip, 90 μL of LMPA (0.5%) were added as a third layer, spread out with the new coverslip, and allowed to harden at 4 °C for 5 min.

The slides were then submerged in a cold lysis solution with a pH of 10 (2.5 M NaCl, 100 mM EDTA, 10 mM Tris pH 10, 1% Triton X-100, 10% DMSO) for at least an hour at refrigerator temperature. After lysis, the slides were placed for 30 min in an electrophoresis tank with a horizontal gel filled with cold (4 °C) alkaline electrophoresis buffer (300 mM NaOH, 1 mM EDTA, pH > 13) in order to unwind the DNA. The following settings were engaged in order to perform electrophoresis: 4 °C, 25 V, and 300 mA for 30 min.

To avoid additional DNA damage, each stage was conducted in complete darkness. After the electrophoresis step, the slides were neutralized three times with 400 mM Tris-HCl (pH 7.5) and finally rinsed in cold distilled water. The slides were stained using 50 μL of ethidium bromide (20 g/mL). The dyed DNA creates a comet-like appearance when observed under a fluorescence microscope (AxioImager Z1, Carl Zeiss, Jena, Germany).

### 2.6. Comet Scoring in AmE-711 Honey Bee Cells

**Figure 1:**
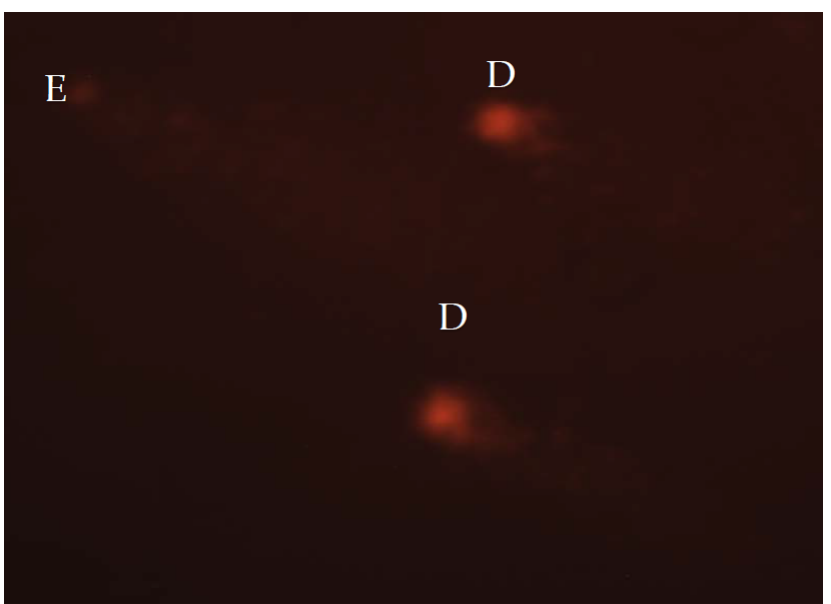
Highly damaged nucleoids stained with ethidium-bromide and analysed under the fluorescent microscope, at 400× magnification. D—high-level damage nuclei; E—total damaged nuclei.

Fluorescence microscope (AxioImager Z1, Carl Zeiss; excitation filter, 515–560 nm; emission filter, 590 nm, Jena, Germany) was used for cell examination. For visual scoring of the comets, Anderson et al.’s [66] method was applied, while Collin’s [67] formula was used to calculate total comet score (TCS). Fifty nuclei from each replicate slide were scored, leading to a total of 100 nuclei for each group. The same operator performed visual scoring of all nuclei. The five classes (A–E) were used to categorize comets. The nuclei without damage are assigned to class A; nuclei with low-level damage to class B; medium-level damaged nuclei were classified to class C; high-level damage nuclei to class D; and total damaged nuclei, also known as “hedgehog comets” were assigned to class E (Figure 1). The formula, TCS = 1 × B + 2 × C + 3 × D + 4 × E, was used to calculate TCS.

### 2.7. Statistical Methods

Shapiro–Wilk’s test was used to determine the normality of the data. When the data had normal distribution (Shapiro–Wilk’s test, *p* > 0.05), one-way ANOVA was used to compare the groups, followed by Tukey’s test. Mean ± standard deviation (mean ± SD) was used to present the data. The criteria for significance were as follows: *p* < 0.05, *p* < 0.01 and *p* < 0.001. The analyses were carried out with GraphPad Prism 7.0. (GraphPad Software, San Diego, CA, USA).

## 3. Results

### 3.1. Trypan Blue Exclusion Assay

Cell viability was calculated in the groups treated with 10, 100, and 1000 g/mL of thymol, as well as in the negative control group. Each group’s cell viability was greater than 85%. As a result, all experimental groups had stable levels of cytotoxicity, which demonstrated that the design is suitable for genotoxicity analyses.

### 3.2. Potential Genotoxic Effect of Thymol in the Comet Assay

**Figure 2:**
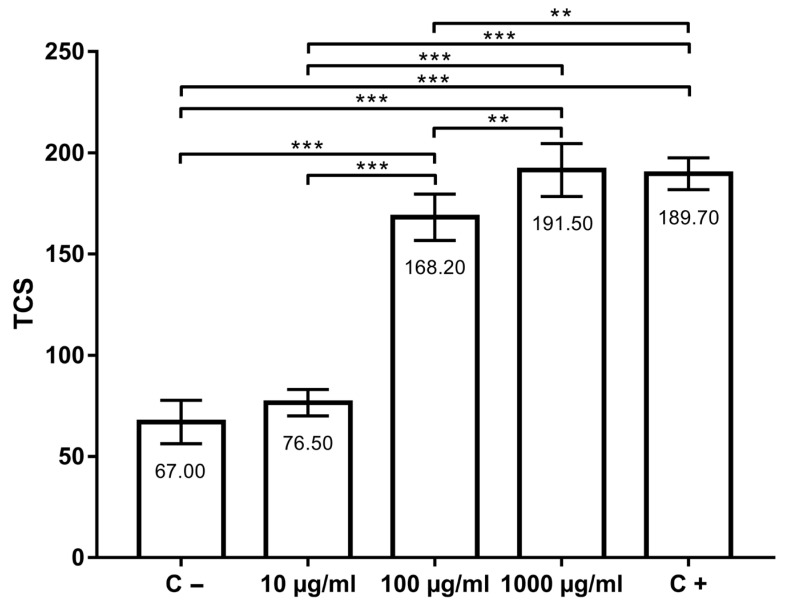
Genotoxic effects of thymol at the concentrations 10, 100 and 1000 µg/mL after 30 min of incubation at 32 °C compared with positive (C+) and negative (C−) controls. **, *p* < 0.01; ***, *p* < 0.001.

Total comet score (TCS) values (indicators of primary DNA damage) are shown in Figure 2. In the negative control group, TCS was 67.00 ± 10.70, while in the positive control group, TCS was 189.70 ± 7.87. TCSs in groups treated with thymol were 76.50 ± 6.54 (10 µg/mL), 168.20 ± 11.44 (100 µg/mL) and 191.50 ± 13.07 (1000 µg/mL). DNA damage in AmE-711 cells treated with 10 µg/mL of thymol was not higher (*p* > 0.05) when compared to the negative control group. However, the concentration of 100 and 1000 µg/mL, as well as 100 µM H_2_O_2_ (positive control)_,_ increased DNA damage in comparison to the negative control (*p* < 0.001). When the groups treated with thymol (10, 100, and 1000 µg/mL) were compared to each other, a significant difference was found between them (*p* < 0.01). DNA migration in positive control was significantly higher than in the group treated with 10 µg/mL (*p* < 0.001) and 100 µg/mL (*p* < 0.01) of thymol. There were no significant difference between the positive control and the group treated with 1000 µg/mL of thymol.

### 3.3. Potential Antigenotoxic Effect of Thymol in the Comet Assay

**Figure 3:**
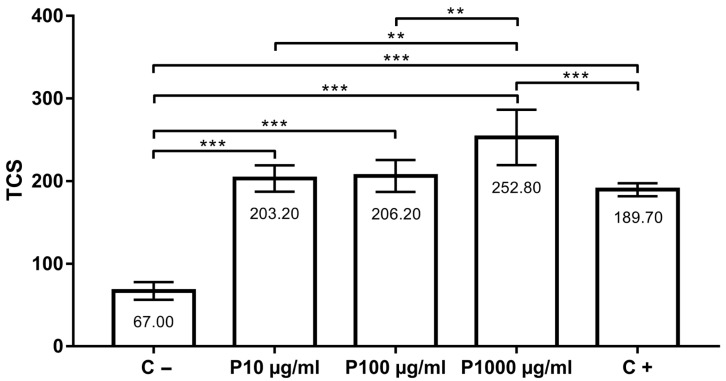
Antigenotoxicity effects of 10, 100 and 1000 µg/mL of thymol in comparison to positive (C+) and negative (C−) control. **, *p* < 0.01; ***, *p* < 0.001.

The assessment of the potential antigenotoxic effect of tested concentrations (groups P10 µg/mL, P100 µg/mL and P1000 µg/mL) showed that thymol did not protect cells from damage induced by H_2_O_2_. Moreover, thymol in the concentration of 1000 µg/mL significantly potentiated the DNA migration in comparison to the positive control (*p* < 0.001). When the groups co-treated with thymol and 100 µM H_2_O_2_ were compared to each other, a significant difference was found between P10 and P1000 µg/mL (*p* < 0.01) and P100 and P1000 µg/mL (*p* < 0.01). TCS in the positive control was 189.70 ± 7.87, while DNA damage in the negative control group was 67.00 ± 10.70. In groups co-treated with thymol (10, 100 and 1000 µg/mL) and 100 µM H_2_O_2_, TCS values were 203.20 ± 15.99, 206.20 ± 19.42 and 252.80 ± 33.4, respectively (Figure 3).

## 4. Discussion

Thymol (chemically known as 2-isopropyl-5-methylphenol and 5-methyl-2-isopropylphenol) is a natural monoterpenoid phenol, with an authentic smell and colourless crystalline structure. It is an isomer with carvacrol and an active ingredient of essential oil extracted from *Thymus vulgaris*, commonly known as thyme [68]. Several medical indications have been reported for thymol, including disorders of the respiratory and gastrointestinal systems, [69], as well as dental diseases such as caries [70,71]. Additionally, positive effects and indications of thymol are found in dermatology for wound healing, which can be used for the development of novel wound dressings [72]. Antioxidant [34], anti-inflammatory [73,74], antifungal [75,76] and antimicrobial activities [77] of thymol are also reported. However, thymol has certain harmful effects on bees that have been demonstrated in both laboratory and field-based studies. In the research of Ellis and Baxendale, [44], thymol was the most toxic monoterpenoid evaluated in a laboratory experiment when used as a fumigant on bees. Moreover, thyme essential oil was the only essential oil that caused bee mortality after their exposure to topical, vapour, and possibly oral treatments in the study conducted by Damiani et al. [23] in which four different botanical oils were investigated. Under field conditions, thymol adversely affected colony development [25,45], the queen [49,50] and the behaviour of bees [46,47,48]. 

The honey bee *A. mellifera* is thought to be the most negatively impacted by the honey bee mite *V. destructor*. The mite, which is present almost everywhere in the world, is the main factor contributing to the decline of honey bee colonies [78]. Due to its role as a mechanical or biological vector of pathogen microorganisms, notably viruses, with the potential to exacerbate pre-existing infections, and its feeding on the fat body and haemolymph of adult and growing honey bees, *Varroa* poses major health risks to its hosts. [9,78,79]. With this in mind, acaricides, whether synthetic or natural-based, must be used continuously in order to control the mite infestation. Thymol is known as a potent acaricide regardless of whether it is used alone or in combination with other biotechnical control methods [80,81]. For use in beekeeping, thymol is usually obtained synthetically and there are many preparations based on this substance [56]. Thymol can cause severe DNA damage through several mechanisms including the induction of reactive oxygen species, which entails an increase in oxidative stress and mitochondrial dysfunction or nuclear factor of activated T-cells (NFAT-2) pathway [82]. To date, various cells (human lymphocytes, V79 Chinese hamster lung fibroblasts, mouse cortical neurons, and peripheral-blood mononuclear cells) have been used for the investigation of the cytotoxic, genotoxic, and antioxidative potentials of thymol [83,84,85,86].

To our knowledge, this is the first study where AmE-711 cells were used to assess the genotoxicity of thymol. The AmE-711 cell line is the first continuous honey bee cell line successfully used in a genotoxicity study [65] and could be applicable in studies of honey bee development, genetics, physiology, pathophysiology and toxicology [59]. The studies carried out on thymol’s genotoxicity in several cell systems gave contradictory results; some of them suggest that this natural plant compound has antigenotoxic potential, while others revealed damaging effects including cytotoxicity and genotoxicity [54,57,58]. In this study, the cytotoxicity of thymol was assessed using the Trypan blue exclusion test, a method suitable as an indicator of cell viability and determination of cytotoxicity. All tested concentrations of thymol exerted cytotoxic effects acceptable for further genotoxicity investigation. Our research indicates that thymol possesses genotoxic potential since the tested concentrations of 100 and 1000 µg/mL significantly (*p* < 0.001) increased primary DNA damage in comparison to the negative control. Significant differences found between increasing concentrations of thymol indicate its concentration-dependent genotoxic effect in AmE-711 continuous cell line. Moreover, DNA damage in group treated with 1000 µg/mL of thymol was even higher than in positive control group. Concordant results were obtained by Hameed et al. [87] who reported the genotoxicity potential of *T. vulgaris* extract against *Salmonella typhimurium*, based on increased tail moments at the concentrations of 1 and 5 mg/mL. The authors found an overall dose effect proven by a proportional increase in extract concentration and genotoxicity effect. The experiment performed on gastric adenocarcinoma cells shows thymol’s in vitro anticancer potential with significant cytotoxic, genotoxic, and apoptotic effects [54]. The genotoxic potential of thymol was reported in research carried out by Radakovic et al. [88] where thymol was used as a positive control in experiment done on cultured human lymphocytes. The results of the present investigation corroborate that finding.

A lack of significant difference between the positive control and 1000 µg/mL of thymol in this genotoxicity experiment inspired us to determine whether thymol can mitigate the genotoxic effects of H_2_O_2_ on the AmE-711 continuous cell line. The obtained results show that thymol did not reduce primary DNA damage caused by H_2_O_2_. Moreover, thymol in the concentration of 1000 µg/mL co-treated with 100 µM H_2_O_2_ significantly (*p* < 0.001) potentiated DNA damage compared to the positive control (cells treated only with 100 µM H_2_O_2_). These findings indicate that thymol potentiated the genotoxic effect of hydrogen peroxide in the AmE-711 continuous cell line. Therefore, we can conclude that thymol has significant genotoxic effects and could be used as a positive control in studies of (anti)genotoxicity on the AmE-711 honey bee cell line. Furthermore, newly synthetized thymol (thymol β-D-glucoside) significantly increased DNA damage in the HT-29 cell line even at non-cytotoxic concentrations [55], which is in accordance with our results. In contrast with our research, Thapa et al. [89] found that thymol in the concentration of 12.5 ppm had exerted a genoprotective effect in HT-29 adenocarcinoma cells. Additionally, standard doses of thymol used in food packaging (0–250 μM) were evaluated for the potential in vitro genotoxicity and showed no damaging effect in mammalian cells [90]. Furthermore, an investigation carried out in the human colon carcinoma cell line Caco-2 using the standard Comet assay revealed that thymol in the concentration range of 0–250 μM did not have any effects after 24 and 48 h of treatment [58]. 

## 5. Conclusions

This study demonstrated the genotoxic effects of thymol in the AmE-711 honey bee continuous cell line for the first time. One should be careful with the preventive, uncontrolled and an excessive use of thymol in beekeeping practice considering the obtained results in an alkaline Comet assay in our study, which suggest a genotoxic effect of thymol, as well as the absence of its antigenotoxic effect against H_2_O_2_-induced DNA damage. Moreover, our findings are even more significant considering that thymol is regarded as a safe substance which is widely used for *Varroa* control in beekeeping sector worldwide. Knowing that this is the first study on the genotoxic effects of thymol performed on honey bee cells, further investigations are necessary to confirm these findings and elucidate the role of DNA damage induced by thymol.

## Data Availability

The data presented in this study are available on request from the corresponding author. The data are not publicly available due to the excessive data size.
